# Competition Stress Prolongs Exercise Recovery in Female Division I Collegiate Soccer Players

**DOI:** 10.3390/sports13120454

**Published:** 2025-12-16

**Authors:** Courtney D. Jensen, Ryann L. Martinez, Nathaniel J. Holmgren, Alexis C. King

**Affiliations:** Department of Health and Exercise Science, University of the Pacific, Stockton, CA 95211, USA; r_martinez22@u.pacific.edu (R.L.M.); n_holmgren@u.pacific.edu (N.J.H.)

**Keywords:** collegiate soccer, allostatic load, training load, recovery

## Abstract

This study examined the effect of competition stress on recovery time in female collegiate soccer players. Thirty NCAA Division I athletes were monitored over 35 consecutive days using Polar Team Pro wearable devices, which captured exercise duration, distance covered, energy expenditure, sprint count, speed, heart rate, training load, and recovery duration. Data were collected across 20 practices and 7 competitions, totaling 845 observations. Linear regression was used to assess whether formal competition independently influenced recovery duration, controlling for time of day and workload variables. Athletes averaged 20.1 ± 1.1 years of age. Across all sessions, the mean exercise duration was 59.5 ± 38.7 min, with an average distance of 2.6 ± 2.1 km, and energy expenditure of 387.2 ± 283.5 kcals. Recovery duration was significantly longer after competition (51.3 ± 59.6 h) compared to practice (13.0 ± 15.8 h, *p* < 0.001). The regression model indicated that formal competition predicted an additional 51 h of recovery time (β = 50.540; *p* < 0.001), independent of physical workload. Recovery following formal competition is significantly prolonged, holding multiple components of workload constant. These findings offer novel insights into female athlete recovery and highlight the importance of sex-specific approaches in sports science.

## 1. Introduction

In the demanding world of collegiate athletics, effective conditioning and recovery are critical to both performance and injury prevention. Soccer, in particular, places unique physical and psychological demands on athletes. Elite players cover 9.5 to 11 km during a 90 min match, with approximately 70% of the activity consisting of low-intensity movement such as walking or jogging and the remaining 30% involving high-intensity running and sprinting [1,2,3]. Although sprinting only accounts for 5–10% of total distance covered, it represents a key component of explosive play, contributing to critical moments in match performance [3]. To meet these physical demands, coaches often use periodization models and monitor training load (TL) to optimize athlete preparedness and recovery. TL can be attributed to duration, intensity, and volume of training and is commonly divided into external and internal load components [4,5]. External load refers to activities prescribed by the coach (drills, 5v5 small-sided games) while internal load reflects the physiological and psychological stress imposed on the athlete [6]. Properly balanced TL is essential, as both insufficient and excessive loading can increase the risk of overreaching or overtraining, which may manifest as decreased performance, heightened fatigue, mood disturbances, and impaired recovery [6,7,8].

Recovery from both training and match play is essential to restoring homeostasis, facilitating muscle repair, and preventing injury. While acute fatigue can be evident for several hours to days following exercise, inadequate recovery periods, particularly when paired with elevated training loads, have been implicated in increased injury risk [2,6,9]. Importantly, although practice sessions may replicate the physical demands of competition, they often fail to reproduce the psychological intensity and emotional stress experienced during formal competition [10]. This psychological stress may serve as an underrecognized contributor to recovery delays, aligning with research suggesting that stress-induced elevations in allostatic load can impair physiological recovery processes [11].

Despite this growing recognition of psychological contributors to recovery, few studies have directly examined the impact of competition-specific stress on objective recovery metrics in collegiate athletes. Most research has focused on physical workload as the primary driver of recovery needs, often overlooking the psychological stressors unique to formal competition, such as performance anxiety, pressure to win, and crowd dynamics [12,13,14,15]. Furthermore, most existing research has focused on male athletes, with limited attention to how female athletes may uniquely experience or respond to training and competition-related stress [16,17]. Given the growing emphasis on equity in sport science and the physiological and psychological distinctions that may influence training outcomes, this gap warrants deeper investigation. The gap is particularly important because sex-based physiological differences, such as hormonal fluctuations, autonomic nervous system regulation, and muscle damage biomarkers, can affect both stress perception and recovery kinetics [18,19]. For example, studies have shown that estrogen may offer protective effects against muscle damage, but fluctuating hormone levels across the menstrual cycle may also contribute to variations in fatigue, soreness, or psychological stress response [18,19]. These distinctions suggest that recovery profiles observed in male athletes may not fully generalize to females, underscoring the need for dedicated investigation in women’s sport contexts.

While this study did not directly assess psychological stress through self-report or biomarker data, the design was intended to evaluate whether recovery time differs between training and competition sessions under otherwise comparable physical workload conditions. As such, although this study does not quantify psychological stress directly, it seeks to provide an initial foundation for understanding how competition contexts may contribute to prolonged recovery. The use of wearable technology, such as the Polar Team Pro system, provides an opportunity to collect high-resolution, objective data on training load, heart rate, and recovery metrics over time. These devices offer real-time data on physical workload, cardiovascular response, and recovery duration, enabling researchers to isolate variables that were previously difficult to quantify in field settings.

Understanding how competition influences recovery independent of physical workload is critical to developing training strategies that promote long-term athlete health and peak performance. Despite a growing female athlete population, women remain underrepresented in sports science research, particularly in studies of recovery physiology. The purpose of this study was to address this gap and determine the effect of competition stress on recovery time in female collegiate soccer players, independent of physical workload. By comparing the impact of recovery duration following formal competition versus training under otherwise similar workload conditions, this research aims to inform evidence-based recovery strategies that better account for both physiological and psychological stressors. Ultimately, these findings may support coaches, athletic trainers, and sports scientists in designing training programs that optimize performance while minimizing injury risk and burnout in female athletes.

## 2. Materials and Methods

### 2.1. Participants

Thirty female NCAA Division I soccer players (mean age 20.1 ± 1.1 years) were recruited from the women’s soccer program at a private university in Northern California. Athletes were active roster members with prior starting experience in NCAA Division I competition. The cohort included 10 defenders, 12 midfielders, and 8 forwards. All participants provided written informed consent prior to participation. This study was approved by the Institutional Review Board at the University of the Pacific. Athletes were eligible if they were active roster members, medically cleared to participate, and injury-free at the start of data collection.

### 2.2. Study Design

This observational cohort study was conducted over 35 consecutive days during the competitive season. Athletes participated in their regular training and competition schedules under the supervision of their coaching staff. Data were collected during 20 practice sessions and seven formal competitive matches, resulting in 845 observations. The typical training week included four practice sessions, a strength training session, and one to two competitive matches. There was one full rest day during each of the seven weeks. Training sessions lasted approximately two hours and alternated between tactical drills and conditioning-focused activities. Athletes were not tracked during strength training sessions. Prior to practices and games, each athlete was fitted with a Polar Team Pro device (Polar Electro Inc., Bethpage, NY, USA), which was worn in a chest strap harness. The Polar system combines GPS and heart rate telemetry to capture external and internal workload metrics. Recorded variables were duration of exercise (minutes), total distance covered (kilometers), estimated energy expenditure (kilocalories), number of sprints performed, average and maximum speed (km/h), average and maximum heart rate (beats per minute), training load and cardio load (arbitrary units calculated by proprietary Polar algorithms), and recovery duration (hours) as calculated by Polar software (v3.1.1). Although a wide array of physiological data was captured, this study did not include direct assessments of psychological stress, such as validated anxiety questionnaires or stress-related biomarkers (e.g., cortisol). Therefore, while differences in recovery may reflect a combination of physical and psychological factors, the latter were not explicitly measured.

### 2.3. Data Collection Protocol

Prior to the start of the study, all devices were inspected, charged, and updated with the latest firmware. Each player was assigned an individual Polar device to ensure consistency across sessions. During an initial orientation session, participants were fitted to confirm device placement and comfort. On each day of data collection, devices were distributed to players approximately 30 min prior to the start of warm-up. Athletes wore the devices during all soccer-related activities, including pre-session warm-ups, practice drills, scrimmages, and match play. Following each session or competition, research staff retrieved the devices. Devices were then connected to the Polar Team Pro docking station for data upload. Session metadata (date, time of day, session type [practice or competition]) were logged manually and verified with the coaching staff. Observations were reviewed for completeness and consistency (e.g., missing heart rate or device malfunction). Sessions with incomplete data were excluded. All observations were merged into a master dataset, which included individual athlete identifiers, session type, and all recorded workload and recovery variables. Time of day was categorized into morning, afternoon, and evening sessions to control for circadian variation. Polar’s proprietary recovery duration algorithm, which integrates training load, heart rate response, and estimated energy expenditure, was used as the primary outcome measure. Three players (all midfielders) did not participate in any games during the study period; they were eliminated from analysis. The remaining 27 players (comprising 818 observations) were considered the study sample.

### 2.4. Statistical Analysis

Each player’s mean performance for each variable was calculated across the 20 practice sessions and across the 7 games. The primary outcome was recovery duration (hours). Workload variables served as predictors of recovery. Descriptive statistics are presented for all variables. Paired-samples t-tests compared practice sessions to games in recovery duration and all workload variables. As all variables may be relevant for understanding athlete workload, correlations between recovery and all workload variables were calculated. Following this, stepwise linear regression models were used to identify the strongest predictors of recovery duration in practice and game settings separately. The variables selected by stepwise regression were then tested together to estimate the amount of variance in excess recovery duration associated with games that workload could account for. Lastly, a series of simple linear regressions tested how incremental increases in performance variables contributed to recovery in practice and in competition. The threshold for statistical significance was set at *p* < 0.05. All analyses were conducted using SPSS version 29.0 (IBM, Chicago, IL, USA).

## 3. Results

Twenty-seven NCAA Division I female collegiate soccer players (mean age 20.2 ± 1.0 years) were included in the analysis. Across all 818 observations, the mean duration of exercise was 59.9 ± 39.1 min, with an average total distance covered of 2.6 ± 2.1 km and estimated energy expenditure of 387.8 ± 287.2 kcal. Athletes performed an average of 6.1 ± 8.0 sprints per session, maintained an average speed of 2.8 ± 1.2 km/h, and reached a maximum speed of 21.1 ± 7.1 km/h. Average heart rate was 132.3 ± 23.8 bpm, and maximum heart rate was 178.3 ± 31.9 bpm. Polar-calculated training load was 71.6 ± 60.0 and cardio load was 77.6 ± 62.9. Recovery duration was 13.1 ± 16.1 h following practice sessions and 51.3 ± 59.6 h following competition.

Comparing individual player means across practice sessions (each player’s individual average values across all practices) to their mean values across all competitions (each player’s individual average performances across all competitions), practice values were significantly lower in most comparisons. During competitions, exercise duration was 186.7% longer (*p* < 0.001), distance covered was 130.2% greater (*p* < 0.001), athletes completed 280.5% more sprints (*p* < 0.001), achieved 20.6% higher maximum speed (*p* < 0.001), had 6.1% higher maximum HR (*p* = 0.008), expended an estimated 144.5% more kcals (*p* < 0.001), and recorded 124.3% greater training load (*p* < 0.001) and 139.4% greater cardio load (*p* = 0.001). Differences between average HR (*p* = 0.058) and average speed (*p* = 0.264) did not reach significance. Recovery duration was 211.8% longer following games than it was after practices (*p* < 0.001). Descriptive and group comparison data are presented in Table 1.

In practice settings, significant correlates with exercise recovery were average HR (*p* = 0.002), maximum HR (*p* < 0.001), training load (*p* < 0.001), cardio load (*p* < 0.001), and estimated energy expenditure (*p* = 0.005). The stepwise regression (R^2^ = 0.681) identified both cardio load (β = 0.255; *p* < 0.001) and training load (β = 0.268; *p* = 0.005) as the only significant predictors in the final model. In competition settings, significant correlates with exercise recovery were average HR (*p* < 0.001), total distance (*p* < 0.001), average speed (*p* < 0.001), maximum speed (*p* = 0.012), number of sprints (*p* = 0.006), training load (*p* < 0.001), cardio load (*p* < 0.001), and estimated energy expenditure (*p* < 0.001). The stepwise regression (R^2^ = 0.718) identified estimated energy (β = 0.150; *p* < 0.001) as the only significant predictor.

Games had 32.8 ± 45.8 h more recovery than practices, the training load was 70.1 ± 72.6 higher, cardio load was 83.8 ± 69.6 higher, and athletes expended an estimated 425.5 ± 265.3 additional kcals. Using excess recovery duration as the dependent variable, stepwise regression found the excess in estimated energy expenditure to be the only significant predictor (R^2^ = 0.718; *p* < 0.001; 95% CI of β = 0.111, 0.185). Owing to multicollinearity, the other two were insignificant (*p* > 0.500). Including all 3 predictors in the model, the R^2^ increased to 0.746 and the adjusted R^2^ was reduced to 0.713, leaving approximately 28.2% of the variance in excess recovery unaccounted for.

**Figure 1:**
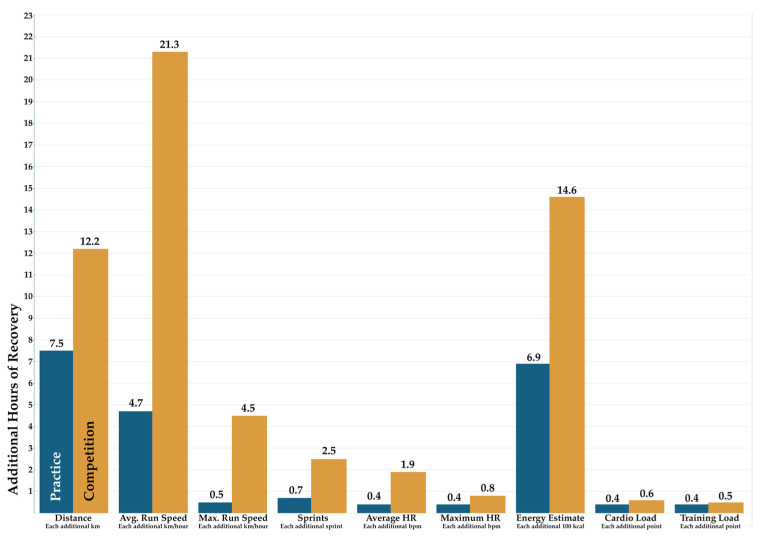
Impact of Training Load Variables on Recovery Time: Practice vs. Competition. Additional hours of recovery required per unit increase in training load metrics for practice (blue) vs. competition (orange).

**Figure 2:**
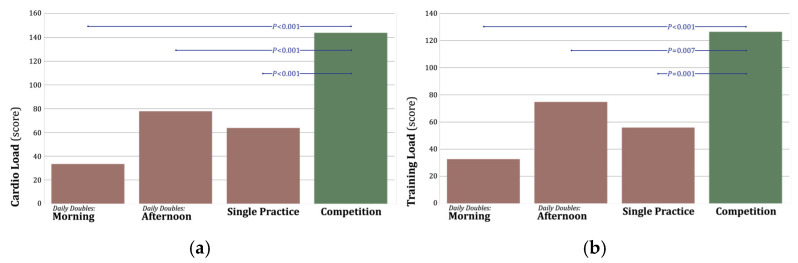
Cardiovascular and Training Load Across Training Phases. (**a**) Cardiovascular load was significantly higher compared to morning daily double practices (*p* < 0.001), afternoon daily doubles (*p* < 0.001), and single-day practices (*p* < 0.001). (**b**) Training load was significantly higher compared to morning daily double practices (*p* < 0.001), afternoon daily doubles (*p* < 0.007), and single-day practices (*p* < 0.001).

Simple linear regression models also identified the effect of incremental increases in all workload variables on recovery duration in practices and in games. Each additional km of distance run predicted a 7.5 h increase in recovery duration after practices (*p* = 0.064; 95% CI of β = −0.5, 15.5) and 12.2 h after games (*p* < 0.001; 95% CI of β = 6.6, 17.8) (Figure 1). Each additional km/hour of average running speed predicted a non-significant 4.7 h increase in recovery duration after practices (*p* = 0.063; 95% CI of β = −0.3, 9.7) and a 21.3 h increase after games (*p* < 0.001; 95% CI of β = 10.6, 32.0). Each additional km/hour of maximum running speed predicted a non-significant 0.5 h increase in recovery duration after practices (*p* = 0.329; 95% CI of β = −0.5, 1.6) and a 4.5 h increase after games (*p* = 0.012; 95% CI of β = 1.1, 8.0). Each additional sprint performed predicted a non-significant 0.7 h increase in recovery duration after practices (*p* = 0.310; 95% CI of β = −0.7, 2.0) and a 2.5 h increase after games (*p* = 0.006; 95% CI of β = 0.8, 4.1). Each additional bpm in average HR prolonged recovery duration by 0.4 h after practices (*p* = 0.002; 95% CI of β = 0.2, 0.7) and 1.9 h after games (*p* < 0.001; 95% CI of β = 1.3, 2.5). Each additional bpm of maximal HR prolonged recovery duration by 0.4 h after practices (*p* < 0.001; 95% CI of β = 0.174, 0.556) and 0.8 h after games (*p* = 0.082; 95% CI of β = −0.1, 1.8), although this relationship did not reach significance. Each additional 100 kcals expended predicted a 6.9 h increase in recovery duration after practices (*p* = 0.005; 95% CI of β = 2.2, 11.6) and 14.6 h after games (*p* < 0.001; 95% CI of β = 10.8, 18.3). Each additional point of cardio load prolonged recovery by 0.4 h after practices (*p* < 0.001; 95% CI of β = 0.2, 0.5) and 0.6 h after games (*p* < 0.001; 95% CI of β = 0.4, 0.7) (Figure 2). Each additional point of training load prolonged recovery by 0.4 h of recovery after practices (*p* < 0.001; 95% CI of β = 0.2, 0.6) and 0.5 h after games (*p* < 0.001; 95% CI of β = 0.4, 0.7). These relationships are depicted in Figure 1.

Collectively, these findings suggest that competition imposes significant additional recovery demands beyond what can be attributed to workload alone. It is important to note that, while these findings indicate a strong association between competition and prolonged recovery, the observational nature of this study limits causal inference. While it is possible that unmeasured factors such as psychological stress may contribute to the observed effect, causality cannot be confirmed.

## 4. Discussion

This study is among the few to focus exclusively on female collegiate athletes in a longitudinal field-based design. Given known sex differences in stress response and recovery, this sample offers critical insights into tailoring recovery strategies for women’s sport. This study investigated the impact of competition stress on recovery time among female collegiate NCAA Division I soccer players, using wearable technology to capture both workload and recovery metrics over 35 consecutive days. Our findings demonstrate that formal competition sessions are associated with prolonged recovery durations compared to training. The duration of recovery in competition was approximately 212% longer, and it was not fully accounted for by the increase in exercise duration and intensity. Unmeasured variables, likely unrelated to training load, contributed to this difference.

These results align with prior research suggesting that psychological stress can exert significant physiological effects on recovery processes. However, psychological stress was not directly measured in this study. While competition is known to increase mental and emotional strain, our inference about its impact on recovery is based on indirect evidence, namely, the prolonged recovery duration after competition under similar physical workload conditions. Future research should integrate direct assessments of psychological stress to better understand this relationship. While much of the literature on training load emphasizes mechanical and metabolic contributors to fatigue and muscle damage, our findings support the growing recognition that contextual stressors, such as competitive pressure, crowd presence, and perceived stakes, may amplify allostatic load and delay recovery [9,13,20,21,22]. This is consistent with the biopsychosocial model of athletic performance, which posits that emotional and cognitive demands can interact with physical workload to shape recovery outcomes [23].

The magnitude of difference observed in recovery duration underscores the importance of accounting for competition-specific stress in training and recovery planning. Although practice sessions in elite environments can replicate many of the physical demands of competition, they may not fully reproduce the psychological stress response [22]. The stark increase in predicted recovery time per 1 km/h increase in average speed during competition relative to training further highlights this interaction between intensity and context.

Although psychological stress is a plausible explanatory factor for the prolonged recovery observed after competition, it was not directly measured in this study. Therefore, any references to psychological contributors should be viewed as hypotheses rather than confirmed findings. Nevertheless, these findings have important practical implications. Coaches, athletic trainers, and researchers should consider incorporating strategies that address both physical and psychological recovery following competition, such as structured recovery days, sleep optimization, nutrition support, and stress-reduction techniques. Moreover, individualized monitoring of recovery status using wearable devices may help detect when athletes require additional time before returning to high-intensity training loads, potentially reducing injury risk and enhancing performance readiness [15,24,25].

This study also contributes to addressing the relative lack of research on female athletes in sports science. Given potential sex-specific differences in stress perception and physiological recovery, our results add valuable evidence supporting the need for tailored approaches in women’s soccer.

### 4.1. Practical Application

The findings from this study offer several actionable insights for sport scientists, coaches, and athletic trainers working with female collegiate soccer players. Recovery duration was significantly longer following competition compared to training, even when controlling for workload variables such as duration, distance, and heart rate. This suggests that coaches should not assume equal recovery needs between session types, even if the physical output appears similar. Additionally, sessions with higher physical loads, such as those exceeding 3.5 km in distance or involving more than 6 sprints, were often associated with recovery durations surpassing 36–48 h. Categorizing training sessions by workload intensity and monitoring recovery time using wearable technology can help guide individualized recovery strategies. Scheduling lighter training or rest following high-load or competition sessions may enhance athlete readiness and reduce injury risk. Finally, while psychological stress was not directly measured, it should be considered in recovery planning, especially in high-stakes or emotionally charged competitions.

### 4.2. Limitations

Several limitations should be noted. First, while Polar Team Pro devices provide validated estimates of workload and recovery, proprietary algorithms for recovery duration may not capture all relevant dimensions of recovery [22]. Second, we did not directly measure psychological stress through validated questionnaires or biomarkers (e.g., cortisol), limiting our ability to definitively attribute extended recovery to psychological factors. Third, our sample was limited to a single NCAA Division I program, which may constrain generalizability to other levels of play or male athletes. Finally, while the observational design captures real-world training and competition patterns, it precludes causal inference. Furthermore, the study did not incorporate subjective or biological markers of psychological stress, such as anxiety surveys or salivary cortisol. As a result, the role of psychological stress remains speculative and should be directly examined in future research.

### 4.3. Future Directions

Future research should combine wearable technology with direct measures of psychological stress, such as self-reported anxiety, salivary cortisol, or heart rate variability, to better disentangle the contribution of psychological and physiological factors to recovery time. Studies evaluating the effectiveness of recovery interventions targeting psychological stress following competition, such as mindfulness training, relaxation protocols, are also warranted. Finally, replication in larger and more diverse cohorts is needed to confirm the robustness of these findings.

## 5. Conclusions

This study demonstrates that formal competition substantially prolongs recovery duration in female collegiate soccer players, independent of measured physical workload. One potential explanation we explored was total distance as a predictor of recovery time, which emerged as a significant factor; however, even after adjusting for distance and other workload measures, the effect of competition remained pronounced. These findings underscore that sufficient recovery is a crucial component of optimal performance and athlete well-being in collegiate athletics. Repeated bouts of incomplete recovery before the next training session or match can contribute to overreaching and elevate the risk of injury. In many cases, limited recovery time may be driven by scheduling constraints inherent to competitive seasons, emphasizing that recovery should be prioritized as a critical element in practice planning rather than viewed as discretionary.

Because the energy demands of a soccer match are highly variable depending on each player’s position, tactical role, and situational demands, individualized recovery strategies may be necessary to maintain optimal performance levels and reduce injury risk. Individual external loads inevitably produce unique internal loads, with varied physiological and psychological impacts on each athlete. Therefore, integrating player-specific monitoring and recovery protocols, including both physical and psychological recovery approaches, may be essential to effective workload management. Further research is warranted to investigate the contribution of psychological stress and competition-related allostatic load to prolonged recovery time. A better understanding of these factors can inform evidence-based interventions that support athlete health, performance sustainability, and resilience throughout the competitive season.

## Figures and Tables

**Table 1:** Descriptives and paired-samples t-test comparisons of exercise recovery and workload variables between training sessions (each athlete’s mean of all practice sessions) and competition (each athlete’s mean of all competitions).

Variables	Total (818 Observations)	Training (27 Observations)	Competition (27 Observations)	Sig.
Duration (min)	59.9 ± 39.1	42.5 ± 1.4	121.7 ± 5.8	**<0.001**
Total Distance (km)	2.6 ± 2.1	1.9 ± 0.3	4.5 ± 2.6	**<0.001**
Energy (kcal)	387.8 ± 287.2	294.5 ± 43.1	720.0 ± 272.0	**<0.001**
Sprints	6.1 ± 8.0	3.7 ± 1.7	14.1 ± 9.8	**<0.001**
Average Speed (kph)	2.8 ± 1.2	2.8 ± 0.4	2.5 ± 1.4	0.264
Max Speed (kph)	21.1 ± 7.1	20.2 ± 2.2	24.4 ± 4.9	**<0.001**
Average HR (bpm)	132.3 ± 23.8	136.9 ± 7.7	128.7 ± 19.8	0.058
Max HR (bpm)	178.3 ± 31.9	177.6 ± 9.4	188.5 ± 19.8	**0.008**
Training Load	71.6 ± 60.0	56.4 ± 8.8	126.5 ± 71.4	**<0.001**
Cardio Load	77.6 ± 62.9	60.1 ± 12.1	143.9 ± 66.7	**<0.001**
Recovery Time (h)	20.7 ± 33.9	15.5 ± 5.7	48.3 ± 46.8	**<0.001**

Values in bold indicate a significant difference (*p* ≤ 0.05) between training and competition.

## Data Availability

Due to participant privacy considerations and institutional review board requirements, the datasets generated and analyzed during the current study are not publicly available. The data presented in this study are available on request from the corresponding author.

## Abbreviations

The following abbreviations are used in this manuscript:

TL	Training Load
HR	Heart Rate
VIF	Variance Inflation Factor
Kmp	Kilometer per Hour
